# Impact of Inoculum Type on the Microbial Community and Power Performance of Urine-Fed Microbial Fuel Cells

**DOI:** 10.3390/microorganisms8121921

**Published:** 2020-12-03

**Authors:** Maria Jose Salar-Garcia, Oluwatosin Obata, Halil Kurt, Kartik Chandran, John Greenman, Ioannis A. Ieropoulos

**Affiliations:** 1 Bristol BioEnergy Centre, Bristol Robotics Laboratory, University of the West of England, Bristol BS16 1QY, UK; Oluwatosin.Obata@newcastle.ac.uk (O.O.); john.greenman@uwe.ac.uk (J.G.); 2 Department of Earth and Environmental Engineering, Columbia University, New York, NY 10027, USA; peziza@yahoo.com (H.K.); kc2288@columbia.edu (K.C.)

**Keywords:** microbial fuel cell, urine, inoculation method, electroactive bacteria, bioenergy

## Abstract

Bacteria are the driving force of the microbial fuel cell (MFC) technology, which benefits from their natural ability to degrade organic matter and generate electricity. The development of an efficient anodic biofilm has a significant impact on the power performance of this technology so it is essential to understand the effects of the inoculum nature on the anodic bacterial diversity and establish its relationship with the power performance of the system. Thus, this work aims at analysing the impact of 3 different types of inoculum: (i) stored urine, (ii) sludge and (iii) effluent from a working MFC, on the microbial community of the anodic biofilm and therefore on the power performance of urine-fed ceramic MFCs. The results showed that MFCs inoculated with sludge outperformed the rest and reached a maximum power output of 40.38 mW·m^−2^_anode_ (1.21 mW). The power performance of these systems increased over time whereas the power output by MFCs inoculated either with stored urine or effluent decreased after day 30. These results are directly related to the establishment and adaptation of the microbial community on the anode during the assay. Results showed the direct relationship between the bacterial community composition, originating from the different inocula, and power generation within the MFCs.

## 1. Introduction

The effects of global warming on everyday life, along with the fossil fuel depletion have fuelled the search for alternative, clean energy technologies. Microbial fuel cells (MFCs) emerge as an environmentally friendly technology where bacteria drive the electricity production from the chemical energy stored in a specific substrate [1,2]. These devices consist of an anodic compartment, where bacteria degrade the organic matter, releasing cations, electrons and other metabolic by-products; this half-cell is physically separated from the cathode chamber, by a semi-permeable membrane. Electrons flow from the anode, through an external circuit, to the cathode, where they recombine with the incoming cations (e.g., protons diffusing through the semi-permeable membrane) to complete the reaction and close the circuit. While in the anodic chamber, oxidation of the organic matter takes place, in the cathode an oxidant, e.g., oxygen, is reduced to produce water. However, the oxygen reduction reaction (ORR) on the cathode is one of the limiting factors of this technology because of the need for a catalyst to accelerate the process [3,4]. So far, great efforts have been made in terms of material science to find a suitable catalyst, which balances high catalytic activity, good long-term stability and low cost [5,6,7,8,9]. Platinum group metals (PGMs) are widely used due to their high catalytic activity. However, their high cost and poisoning limitations make them unsuitable for use in scaled-up systems. Carbonaceous materials such as activated carbon or metal-free catalysts represent a real alternative to PGMs due to their low cost and longevity, which enable real-world implementation of this technology [8,10,11].

In addition to the advancements in designing new and efficient catalysts, the modification of the anode surface has been reported as a suitable way to improve the energy generation from MFCs. Activated carbon is a cost-effective material that increases the specific surface area, which facilitates bacterial attachment and development of a viable electroactive biofilm [12]. Other materials as well as techniques have also resulted in increasing specific anode area and consequently the power performance of the MFCs. One of these techniques is the modification of the anode with a wide variety of conductive polymers such as polyaniline (PANI) [13], polypyrrole (PPy) [14] or poly(3,4-ethylenedioxythiophene)-polystyrenesulfonate (PEDOT-PSS) [15].

Another important element of MFCs is the membrane, which separates the anode and the cathode. Commercial polymer-based membranes, e.g., Nafion or Ultrex, have been commonly used due to their high ionic conductivity and good performance. However, their high cost and moderate long-term performance have steered research into low-cost alternatives, which enable the commercialisation of MFCs [16]. Ceramics represent a broad range of materials with meaningful advantages over conventional polymer-based membranes, for instance, their natural availability, cost-effectiveness and robustness, which reduces the maintenance needed in potential scaled-up systems [17,18]. The recent use of MFCs in remote location opts for low-cost materials with low environmental impact. Another benefit of using ceramic materials is that their properties, such as porosity or ionic conductivity, can be tailored by changing the kilning temperature or doping the raw material [19,20,21].

One of the unique advantages of MFCs is the use of different types of waste as feedstock for microbes, whereby contaminants are broken down for the production of electricity [22,23]. So far, a wide range of waste materials have been used as substrates, e.g., waste from the brewery, dairy or oil refinery industries, food processing wastewater and domestic wastewater, among others [24,25,26]. More recently, the use of neat urine as feedstock in different kinds of bioelectrochemical systems has gained a lot of interest due to its abundance, buffering capacity and high chemical oxygen demand [27,28,29]. This natural waste product has been successfully used for ammonium recovery, powering lights, charging smartphones or powering a microcomputer through MFCs [30,31]. For MFCs, it is well known that the concentration, organic matter loading and bacterial diversity of the inoculum as well as feedstock, affect the power performance. The use of complex substrates with high organic loading usually helps in stablishing a diverse and electrochemically active microbial community on the anode. The feed-rate and shear-stress are also important parameters that need to be optimised in continuously fed systems, as is the temperature, since all these can affect the behaviour of the anodic microbial communities. In terms of pH stability, it is important to use a feedstock with natural buffering capacity, such as urine, in order to avoid adding external pH buffer solutions, which increases cost and complicates maintenance. Thus, the choice of an appropriate inoculum and suitable operating conditions, significantly influences the performance of MFCs [32,33].

At present, research is focused on moving the MFC technology from the laboratory to the field. The real implementation of this technology still poses a challenge especially when the deployment takes place in rural places with low or limited development. As the anodic biofilm dictates power output, it is vital to understand the ecological behaviour of the anodic microbial community under different operating conditions [34]. The influence of factors such as the substrate [35,36], external resistance [37] or anode materials [38], on the microbial community have already been reported. According to previous work, the variation of the operating conditions, substrate or electrode materials strongly affects the anodic microbial community and, therefore, the power performance of MFCs. In order to promote the real implementation of this technology, it is important to explore the behaviour of the microbial diversity under different operating conditions, especially in the start-up phase, which still poses a challenge for the functioning of the system.

The enrichment of the bacterial community of the anodic biofilm is a strong indication of process functionality. However, despite the plethora of bacteria present in MFCs, only those that are capable of electroactive metabolism would directly contribute to electricity generation. As such, bacterial population or diversity increases in MFCs do not often translate to increased power generation. Nevertheless, a synergistic approach to electricity generation has been reported where different bacterial strains work in symbiosis to bring about the degradation of various contaminants thereby providing the needed substrates for the electroactive strains [36].

According to this approach, this work aims to investigate in-depth the effect of using three different types of inocula based on human urine on the anodic microbial community of MFCs and, therefore, its correlation with the power performance of the overall system. All tests were performed in triplicate with a total number of 9 MFCs run simultaneously in continuous mode for 90 days. The results were analysed in terms of microbial diversity and power output but also stability and functionality for long operating processes.

## 2. Materials and Methods

### 2.1. Microbial Fuel Cell (MFC) Set-Up

**Figure 1:**
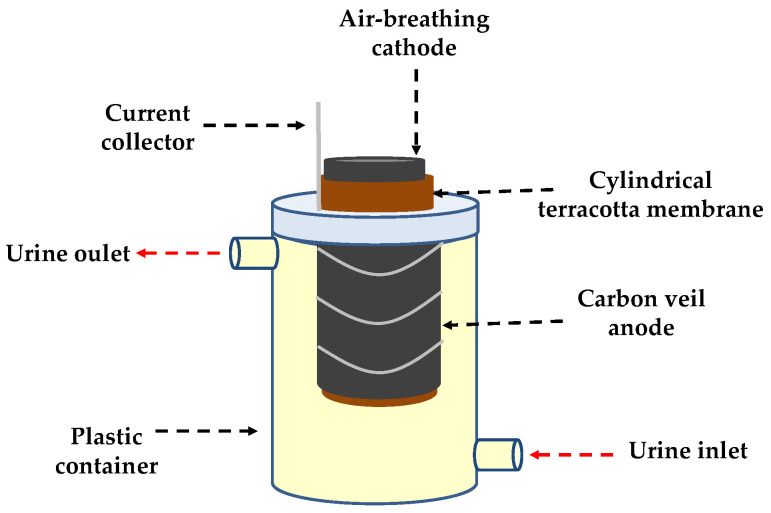
Scheme of the microbial fuel cell (MFC) set-up used in this work.

MFCs were assembled using 5 cm tall terracotta cylinders sealed on one side (Orwell Aquatics, UK). The outer diameter was 2.9 cm, the inner diameter was 2.1 cm, so the thickness of the cylinder wall was 4 mm. The anode consisted of a piece of carbon veil (carbon loading 20 g·m^−2^) of 300 cm^2^ folded and wrapped around the outside wall of the terracotta cylinder. Nickel chromium wire was used for connecting the electrode to the load and data logging apparatus. The cathode was made of a paste of activated carbon and polytetrafluoroethylene (PTFE) hot pressed over a piece of carbon veil (carbon loading 20 g·m^−2^) of 30 cm^2^ [39]. The cathode was placed inside the cylinder and connected to the anode through an external circuit. The cylinder with both electrodes was placed in a plastic container (60 mL working volume) where the outer wall of the terracotta cylinder along with the anode was fully immersed in the anolyte. Anaerobic conditions in the anodic compartment were maintained by using the lid of the plastic container (see Figure 1). A multi-channel data logger (Agilent 34972A, Farnell, UK) was used to continuously monitor the voltage of the systems. The external resistive load was adjusted every 8 days (i.e., whilst maturing) until reaching maximum power, which was achieved across a load of 100 Ω; this remained constant until the end of the experiments.

### 2.2. Inoculation Methods

In this section the procedure employed for each type of inoculum assessed in triplicate is described. All systems were inoculated simultaneously and kept working for 90 days in continuous mode with a total number of 9 MFCs were ran in parallel.

- Method I: The MFCs were inoculated with naturally hydrolysed human urine. Before collection, urine was stored in a tank for at least 48 h, which allowed the hydrolase enzymes to precipitate struvite, increasing the pH to 9.3. The MFC were batch-fed daily for 4 days and then continuously fed with stored urine at a flow rate of 200 mL·day^−1^.
- Method II: In this case, the systems were inoculated with a mixture containing 1/1*_V_*_/*V*_ anaerobic sludge collected from a Wessex Water treatment plant (Saltford, UK) and stored urine (pH: 9.28), which was replenished with fresh solution daily for 4 days. After this period, the MFCs were continuously fed with stored urine at a flow rate of 200 mL·day^−1^.
- Method III: The last inoculum consisted of using a solution containing 1/1*_V_*_/*V*_ effluent from a long-term working MFC and stored urine (pH: 9.29). The solution was replenished daily with a fresh mixture for 4 days, after which period, the systems were continuously fed with stored urine at a flow rate of 200 mL·day^−1^.

### 2.3. Electrochemical Characterisation

The polarisation of the 9-MFCs was performed by using a potentiostat (μAutoLab III/FRA2, Metrohm, The Netherlands) by linear sweep voltammetry (LSV) from open-circuit voltage (OCV) to 0.05 mV at a scan rate of 0.25 mV·s^−1^. The polarisation of the MFCs was performed 4 times during the experiment: on days 15, 30, 60 and 90; subsequently the external load was kept constant. The measurements were performed at a stable open-circuit voltage in a two-electrode configuration where the cathode is connected to the working electrode, the anode is connected to the counter electrode and the reference channel is short-circuited with the counter electrode channel. Polarisation curves of the 9-MFCs were obtained by plotting the cell voltage versus current (V vs. I) whereas power curves were obtained by plotting power versus current (P vs. I).

For a better understanding of the impact of the inoculum type on the anode performance, the electrodes were individually characterised by LSV in a three-electrode configuration at the same scan rate as with the complete cell. The polarisation of the anode was performed connecting the anode to the working electrode, the cathode to the counter and the reference channel to a Ag/AgCl 3M KCl electrode. The measurements were performed between open-circuit potential (OCP) and −0.1 mV vs. Ag/AgCl. For the polarisation of the cathode, the cathode was set as the working electrode and the anode as the counter electrode, keeping Ag/AgCl 3M KCl as the reference electrode.

### 2.4. DNA Isolation, Next-Generation 16S rRNA Amplicon Sequencing and Sequence Data Analysis

Anodic bacterial community as well as the starting materials (sludge and urine) were collected at regular intervals for DNA analysis (T0, T30, T60 and T90). Metagenomic DNA from samples were extracted in duplicates using Dneasy Blood and Tissue kit (Qiagen, Germantown, MD, USA) according to kit protocol. DNA quality and quantity were measured by Nano-Drop Lite spectrophotometer (Thermo Fisher Scientific, Waltham, MA, USA). Bacterial 16S rRNA gene was amplified using universal primers 1055f (ATGGCTGTCGTCAGCT) and 1392r (ACGGGCGGTGTGTAC) [40] and barcoded fusion primers with sequencing adaptors. Polymerase chain reaction (PCR) runs were carried out in a total 25 µL volume containing 0.5 µL of forward primer, 0.5 µL of reverse primer (10 ρmoles/µL), 0.1 µL of MyTaq polymerase (5 u/µL). Other components of the mix are 5 µL of PCR buffer (comprising 5 mM dNTPs, 15 mM MgCl_2_, stabilizers and enhancers), 18.4 µL of molecular grade water (17.4 µL) and 0.5 µL of DNA sample. Negative controls containing 0.5 µL of sterile molecular grade water were included in all cases. The PCR reaction was started with initial denaturation at 95 °C for 3 min, denaturation at 95 °C for 1 min, annealing at 55 °C for 1 min and extension at 72 °C for 1 min, in a total of 30 cycles [41]. The quality and quantity of the 16S amplicon sequence was checked with bioanalyser (Agilent Technologies 2100, Santa Clara, CA, USA). The 16S amplicon sequencing was performed using an Ion Torrent PGM (Thermo Fisher, Bedford, MA, USA) platform with Ion Torrent 318v2 Ion Chip by following manufacturer’s instructions (Ion PGM Hi-Q Sequencing kit, Thermo Fischer, Bedford, MA, USA). All 16S rRNA gene raw sequences have been deposited to Sequence Read Archive (SRA) at the National Center for Biotechnology Information (NCBI) under the accession number SAMN12583022-SAMN12583029. Qiime2 V.2018.11 [42] pipeline was used 16S amplicon data analysis. Quality check and chimera removal of 16S amplicon reads were performed dada2 command. Taxonomic classification was performed by consensus-blast command against Silva ribosomal databases v132 [43].

## 3. Results

### 3.1. Microbial Fuel Cell Performance

**Figure 2:**
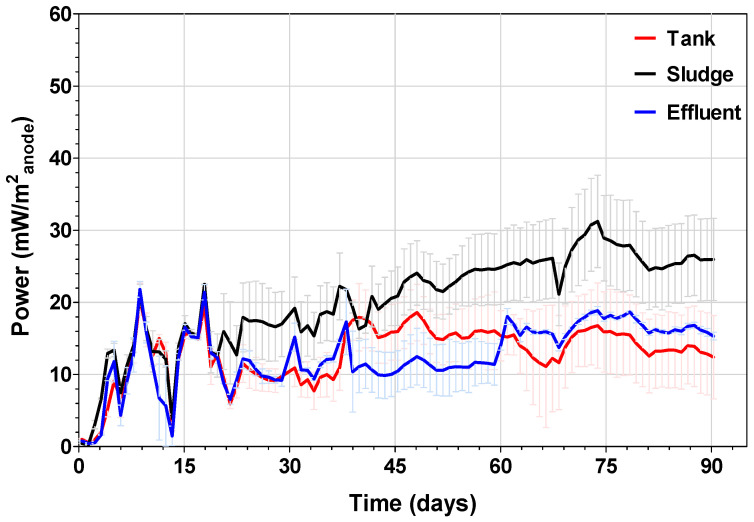
Long-term power performance of the MFCs.

Before characterising the electrochemical performance of MFCs, the stability of power output was investigated in triplicate for 90 days in continuous mode. As explained in the Materials and Methods section, each triplicate was simultaneously inoculated according to the three methods described above. As shown in Figure 2, the power output of all MFCs from all conditions was quite similar since the anodic biofilm had not been completely formed yet. However, after day 20, the power output levels started varying significantly. From then on, MFCs inoculated with sludge outperformed both the MFCs inoculated with stored urine and those with MFC effluent, reaching a maximum value of 31 mW·m^−2^_anode_ (0.93 mW). Once the systems reached steady-state, the MFCs inoculated with sludge maintained stable power production between 25 mW·m^−2^_anode_ and 26.7 mW·m^−2^_anode_ (0.75 and 0.8 mW). The reduction in power observed at certain points was caused by electrical connection artifacts, however the power output completely recovered after these were repaired. In the case of the MFCs inoculated with stored urine and sludge, both triplicates exhibited similar values of power output until day 37, when important differences were observed. From day 37 until day 60, the MFCs inoculated with stored urine outperformed those inoculated with MFC effluent, reaching a maximum value of 18.3 mW·m^−2^_anode_ (0.55 mW). However, after day 60 the power performance of MFCs inoculated with effluent increased above that from MFCs inoculated with stored urine, reaching a maximum value of 19 mW·m^−2^_anode_ (0.57 mW). Subsequently, the mean power output by these systems was 17.7 mW·m^−2^_anode_ (0.53 mW). In the case of the MFCs inoculated with stored urine, the power performance remained stable from day 37 until the end of the investigation. Only a slight reduction in power was observed between days 62 and 65 due to lack of feeding. Once feeding was reinstated, the systems returned to the previous values. These results may be related to differences in the anodic biofilm developed as a result of the different origins. According to the long-term power performance, the use of sludge as bacterial inoculum in urine fed MFCs might promote the development of a more efficient biofilm than using stored urine or effluent from other urine-fed MFCs. When comparing the results obtained using sludge and MFC effluent as inocula, it seems that stored urine reaches steady state faster than those systems inoculated with effluent. However, despite taking longer to adjust, eventually power output was highest. It may be possible that bacteria coming from other MFCs as those contained in the effluent inoculum, need more time to adapt to the new operating conditions but they are able to develop a more efficient biofilm than bacteria present in stored urine.

**Figure 3:**
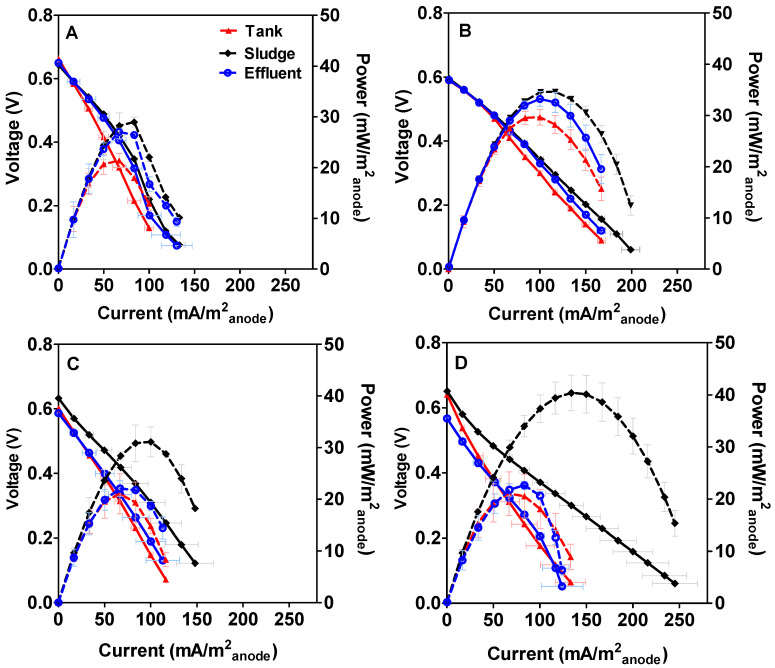
Polarisation and power curves (dashed line) of MFCs inoculated with the different methods after 15 (**A**), 30 (**B**), 60 (**C**) and 90 days (**D**).

**Figure 4:**
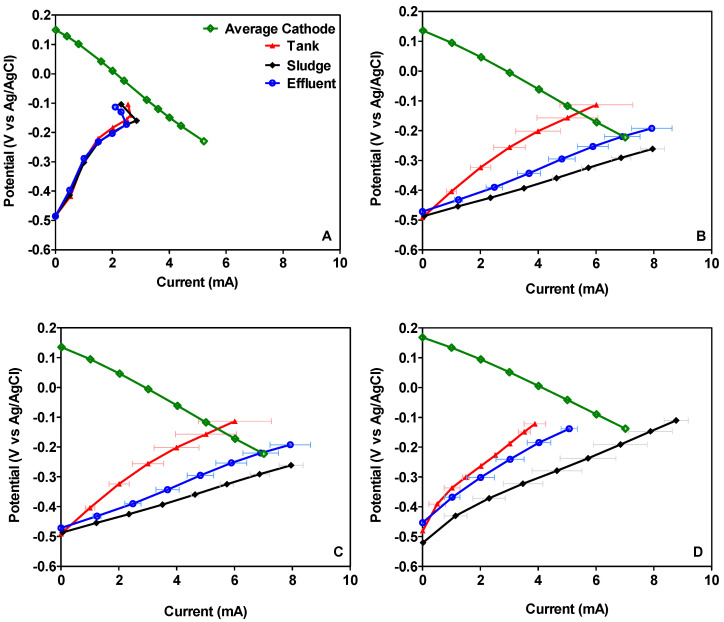
Electrode polarisation of the MFCs after 15 (**A**), 30 (**B**), 60 (**C**) and 90 (**D**) days.

Figure 3 shows the average electrochemical performance of the overall cells after 15, 30, 60 and 90 days working in continuous mode. According to the long-term power output previously discussed, the maximum value was reached by MFCs inoculated with sludge. It was also observed than this value increased over time, reaching a maximum of 40.38 mW·m^−2^_anode_ (1.21 mW) after 90 days. These results are in line with the electrochemical characterisation of the individual electrodes shown in Figure 4, which shows how the anode of the MFCs inoculated with sludge improved its performance over time (see Figure 4D). By contrast, the maximum power output reached by the MFCs inoculated either with stored urine or effluent decreased over time, reaching in both cases the maximum value in the polarisation on day 30. After 30 days, the MFCs inoculated with stored urine produced a maximum power of 29.64 mW·m^−2^_anode_ (0.89 mW), which then decreased by 29% and thereon remained stable until the end (21.04 mW·m^−2^_anode_–0.63 mW). A similar behaviour was observed in the case of the MFCs inoculated with effluent whereby the maximum power output was observed on day 30 (33.25 mW·m^−2^_anode_–0.99 mW), after which maximum power decreased to 22.06 mW·m^−2^_anode_ (0.66 mW), and remained stable until the end of the experiment. These results are in line with the electrochemical characterisation of the anode displayed in Figure 4 where it is noticeable that the anode in MFCs inoculated either with stored urine or effluent show the best performance on day 30 (see Figure 4B). These results may be related to the changes observed in the anodic microbial community from day 30, which showed a significant shift in bacterial community structure.

Table 1 summarises the maximum power output reached by the MFCs inoculated with the different sources over time. The statistical analysis of the results (data not shown) demonstrates that the nature of the different inocula investigated has a significant effect on the power output (*p* < 0.05). As can be seen in Table 1, power output by the sludge-inoculated MFCs greatly increased over time, reaching the highest value after 90 days of operation. By contrast, the maximum power performance of the rest of the conditions investigated is lower, reaching a plateau after 60 days of operation.

### 3.2. Microbial Ecology and Phyla Distribution

**Figure 5:**
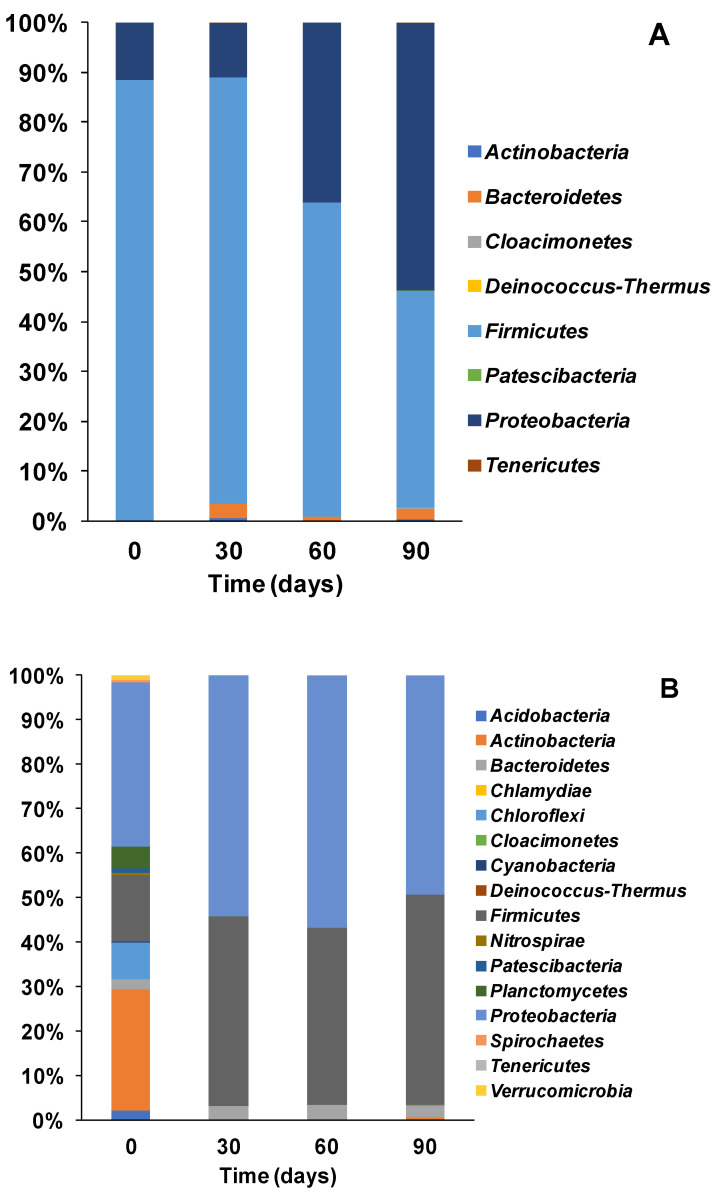

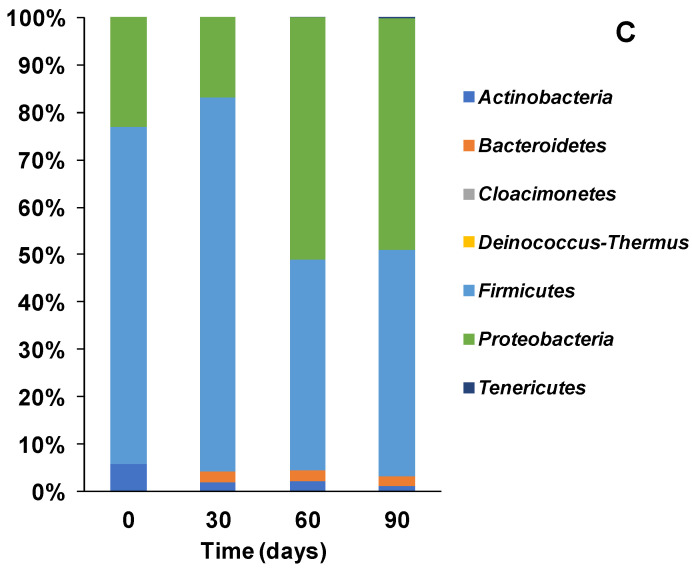
Phyla distribution of the microbial community detected in the stored urine (**A**), sludge (**B**) and MFC effluent (**C**) used as inocula (according to the different methods described earlier).

Analysis of the bacteria community at the phylum level showed that Firmicutes strains were dominant within the bacterial community of the stored urine at the start of the experiment (T0). This dominance was observed up until day 30, where they account for about 90% of the bacterial community (Figure 5A). However, by day 60, the proportion of Firmicutes reduced to ca. 63% as the proportion of Proteobacteria increased (from only 10% at T0 and day 30) to approximately 36%. By day 90, Proteobacteria dominated, making up 54% of the population whilst the proportion of Firmicutes decreased to 45% (Figure 5A). These results highlight a gradual redistribution of the anodic communities, which perhaps is a response to the various biochemical reactions occurring within the MFC reactors over time.

For the sludge inoculated reactors, analysis at the phyla level revealed a diverse community at the start of the experiment, which was dominated by Proteobacteria (37%) and Actinobacteria (27%). By day 30, there was a significant shift in the community structure and with a concomitant decline in diversity resulting in the detection of only 3 main strains namely Proteobacteria (54%)*,* Firmicutes (43%) and Bacteriodetes (3%), compared to more than 10 different phyla at T0. There was also the intriguing disappearance of Actinobacteria which constituted 27% of the community at T0. The shifts in the community structure observed, resulted in the dominance of Proteobacteria and Firmicutes strains from day 30 till the end of the experiment at day 90 (see Figure 5B).

Analysis of the bacterial community of effluents obtained from working MFCs, revealed less diversity at the phyla level compared to the sludge sample. At the start of the experiment T0, only 3 strains were detected, namely Firmicutes (71%) Proteobacteria (23%) and Actinobacteria (6%) (see Figure 5C). By day 30, the proportion of Firmicutes increased to 79% whilst Protoebacteria decreased to 17%, followed by the detection of the phylum Bacteriodetes, whilst the proportion of Actinobacteria remained stable. However, by day 60 the proportion of Proteobacteria increased to 51% making it the most dominant strain until the end of the experiment (49%). The earlier stages of the experiment (T0–T30) saw the dominance of Firmicutes while the latter stages (T60–T90) were dominated by Protoebacteria. This shift in community composition is similar to the results obtained from other inoculation experiments described earlier (see Figure 5).

In general, the results showed a gradual trend of community redistribution leading to a shift of dominance from Firmicutes to Proteobacteria. This observation suggests that members of Firmicutes which were the dominant strains at T0–T30 in all inocula, were important to the various hydrolytic processes at the earlier stages of the experiment. Whereas members of the phylum Proteobacteria, especially for the latter stages (T60–T90) of the experiment, were important for the various biochemical reactions responsible for current generation, in all set ups.

Earlier research has shown the dominance of the two phyla (Protoebacteria and Firmicutes) in MFC systems whether at pilot scale used for the treatment of sewage [44] or operated at lower temperatures [45]. Firmicutes have been previously detected as the dominant strain in glucose fed MFCs, where they are thought to convert carbon into simpler molecules. Being aero-tolerant, Firmicutes have been known for scavenging oxygen, thus providing an enabling environment for facultative anaerobic power producers in MFC [46]. A more recent study reported that members of Firmicutes such as *Clostridium* are able to reduce insoluble iron and generate electricity [47], an indication of their importance in MFCs.

Proteobacteria on the other hand have been detected in many different microbial fuel cell systems including urine fed MFC [48] and MFCs fed with acetate, butyrate and glucose (amongst many other substrates) [49]. Essentially, many of the prominent known electroactive bacteria such as *Shewanella Pseudomonas* and *Geobacter* etc. belong to the phylum Proteobacteria [36,50,51], hence their increased enrichment over time within MFCs (see Figure 5).

### 3.3. Bacterial Composition of Community at Genera Level with Different Inoculations

#### 3.3.1. Bacterial Community Structure and Genus Distribution in Stored Urine Inoculated MFC Reactors

**Figure 6:**
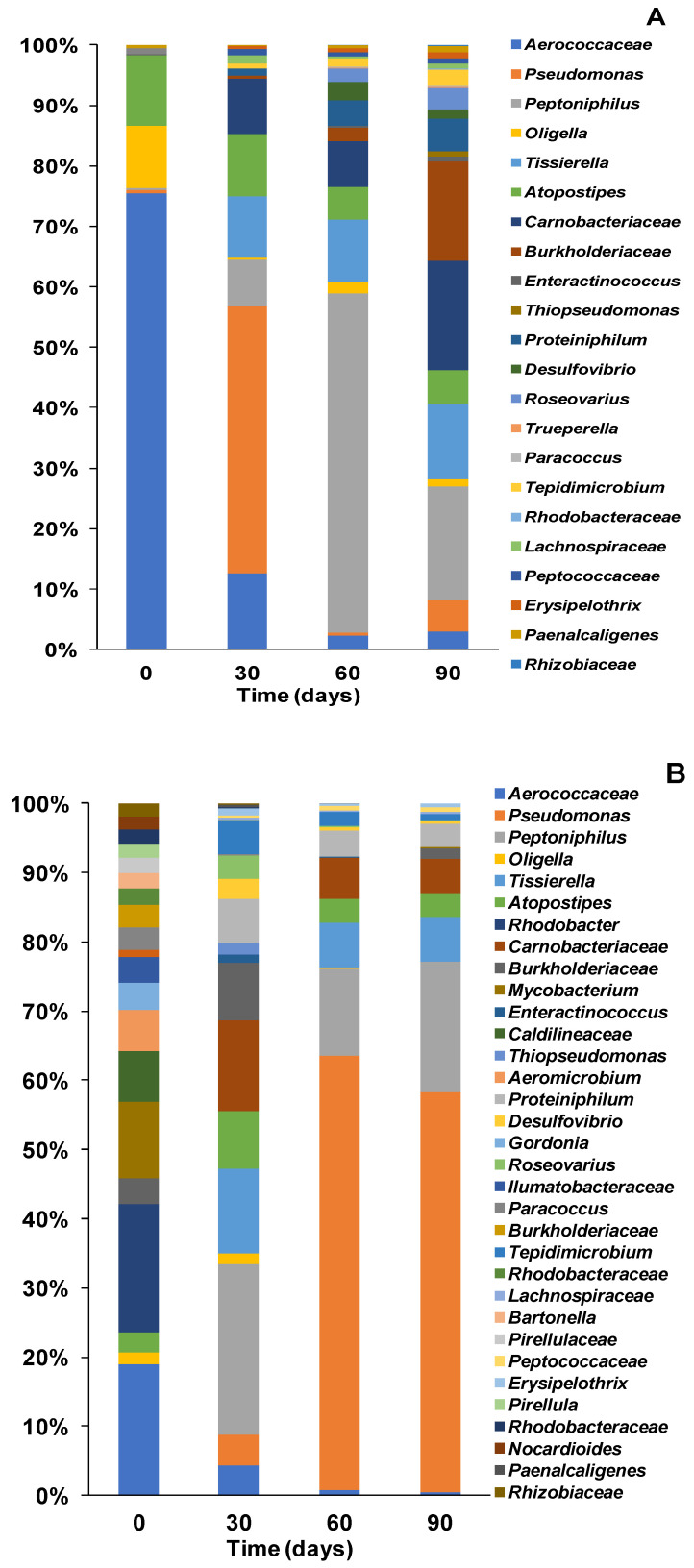

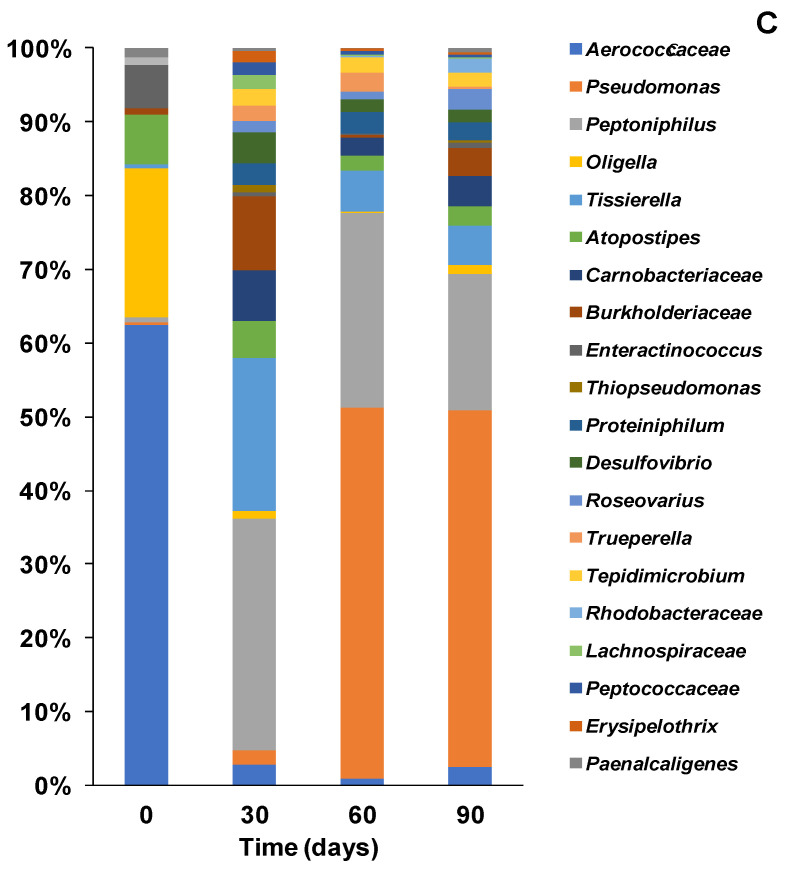
Family and genus distribution of the microbial community detected in the (**A**), sludge (**B**) and effluent (**C**) used to inoculate the MFCs according to the different methods described above.

Analysis of the bacterial community of the stored urine inoculated reactors at family and genus levels highlighted diverse communities within the MFC anode. At the start of the process, the community was dominated by Aerococcaceae (76%) belonging to the phylum Firmicutes while *Atopostipes* and *Oligela* made up 12% and 10% respectively (see Figure 6A). The dominant Aerococcaceae are non-motile, non-spore forming Gram-positive bacteria, which inhabit humans, households and hospital environments [52]. *Aerococcaceae* contain important members including *Aerococcus urinae* which is commonly found in human urine and have been associated with some urinary tract infections [53].

By day 30, there was a shift in the community composition and distribution, resulting in greater diversity. For instance, members of the genus *Tissierella*, which was below detection at the start of the process accounted for 10% of the community while *Pseudomonas* (which was less than 1% at T0), became the dominant species accounting for 44% of the community. Conversely Aerococcaceae which accounted for 76% of the population reduced to only 13% by day 30 while *Atopostipes, Tissierella,* Carnobacteriaceae and *Peptoniphilus* made up 10%, 10%, 9% and 8% respectively.

*Pseudomonas* strains carry out processes using oxidative phosphorylation, a process that enhances current generation in MFCs. *Pseudomonas* species also utilise pyocyanin and phenazine as mediators for electron transfer in MFCs [54].

Between day 30 and 60, a further shift in the community composition was observed with *Peptoniphilus* which previously was only at about 8% becoming the most dominant and accounting for 56% of the community composition. Moreover, the proportion of *Tissierella* and Carnobacteriaceae remained largely stable during the process. By day 90, there were many prominent strains without a single dominant strain within the community. Some of the prominent strains at day 90 include *Peptoniphilus* (19%), Carnobacteriaceae (18%) Burkholderiaceae (16%) and *Tissierella* (13%). The bacterial community at this time was more diverse than previous time points, owning to adaptation and acclimation. Moreover, several changes occurred within the community resulting in the emergence of new strains and reduction in the proportion of previously dominant strains. For instance, members of the genus *Peptoniphilus* which were the dominant strains at T60 (56%), became less dominant at T90 accounting for only 19% of the population. Burkholderiaceae on the other hand, which constituted less than 1% of the community at T0 and T30, and only 2% at T60 became very prominent at T90 accounting for more than 16% of the community. Some members of Burkholderiaceae such as *Rhodoferax* sp. and *Cupriavidus* sp. have been reported to carry out extracellular electron transfer via direct and mediated means [36,55], which may explain their increased enrichment over time in the current study as more direct and indirect electron transfer is required for electricity generation.

#### 3.3.2. Bacteria Community Structure and Genus Distribution in Sludge Inoculated MFC Reactors

Analysis of the bacterial community of the sludge inoculated MFCs revealed a more diverse community with Aerococcaceae and *Rhodobacter* accounting for 19% each respectively at T0 (see Figure 6B). In the first 30 days of the experiment, there was a gradual shift in community distribution resulting in the dominance of the genus *Peptoniphilus*, which made up 25% of the community at T30. There was a further shift in community structure resulting in reduced diversity and the dominance of the genus *Pseudomonas* species at T60 representing 63% of the population and 58% at T90. A general trend was observed in the community structure thus, as the proportion of the originally dominant Aerococcaceae and *Rhodobacter* declined over time, the proportion of *Pseudomonas* increased (Figure 5B). This shift and redistribution of bacterial strains especially after day 30 is perhaps an indication of a period of extensive bio-electrochemical reactions within the MFCs, corresponding to a stage of significant increase in power generation (Figure 2). Moreover, *Pseudomonas* species have been known to contribute to current generation via mediators for electron transfer [44,54] in MFCs. Therefore, their sustained enrichment at the latter stages of the experiments in sludge inoculated reactors’ significantly enhanced power generation.

#### 3.3.3. Bacteria Community Structure and Genus Distribution in Effluent Inoculated MFC Reactors

Figure 6C shows the bacterial community distribution at the genus level over time in the effluent inoculated MFCs. At the start of the experiment (T0), Aerococcaceae was the dominant strain accounting for 63% of the community. The genus *Oligella* was the second most dominant bacteria strain at the stage of the experiment making up just over 20% of the community (Figure 6C). By day 30, there has been a significant shift in community structure which saw the dominant Aerococcaceae go from 63% at T0 to only 3% at T30. However, *Peptoniphilus,* which made up only around 1% at T0 increased to 32% at T30 and remained largely stable over time. *Tissierella* species which was only 1% of the community at T0 had also increased to 21% by day 30. The time when considerable amount of electricity generation occurred in the effluent inoculated MFCs, as shown in Figure 2. *Tissierela* has been reported as an important organism for urine metabolism, which provides the needed carbon source for electroactive communities in urine fed MFCs [36,56]. As the experiment progressed, there was a further shift in the community structure, which resulted in the dominance of the genus *Pseudomonas*, representing 50% of the community while *Peptoniphilus* accounted for 27% (see Figure 6C). The emergence of the dominant *Pseudomonas* at T60 corresponded to a time of increased power generation in the reactors as shown in Figure 1. This is an indication that the *Pseudomonas* species aided electricity generation by the secretion of pyocyanin, which acts as an electron mediator in MFCs [49,57]. Pyocyanin is a known antimicrobial toxin, which may be the reason for the suppression of some of the other species in the mixed community, especially those that were in competition with *P. aeruginosa*. A relative stability was observed within the bacterial community as *Pseudomonas* and *Peptoniphilus* remained the dominant strains accounting for 48% and 19% of the community, respectively.

Overall, *Pseudomonas* species appear to be highly enriched within all MFC reactors in the current study and this might be due to their important contribution to power generation as electron mediators [44].

The establishment of a microbial community in MFCs is naturally dictated by the inoculum, inoculation process and maturing regime [47,49]. The choice of anolyte and substrate and their composition is one of the most important factors that determine the structure, and composition of the microbial community on the anode [58]. Urine fed MFCs have so far been colonised by certain types of bacteria which are quite different from the more conventional and well-known electroactive species such as *Geobacter* and *Shewanella.* Most of the previously reported bacterial communities in urine-fed MFCs were also detected in the current study, including Burkholderiaceae, *Tissierella, Pseudomonas*, Aerococcaceae, *Atopostipes*, *Peptoniphilus*, *Oligella*, *Proteiniphilum* and *Desulfovibrio* among others [36]. There findings suggest that these organisms, although having different metabolic requirements, operate in a synergistic manner to bring about urine degradation and electricity generation recorded in urine-fed MFCs.

## 4. Conclusions

The current study highlights the impact of the inoculum type on the microbial anodic community in urine-fed MFCs and consequently, on power performance. To this end, the MFCs were inoculated with stored urine, sludge and effluent from a working MFC and then fed continuously with neat urine (in triplicate). The results were compared in terms of microbial anodic community and power performance, as well as long-term functionality. Among the different conditions tested, the inoculation of MFCs with sludge seems to promote a more electroactive biofilm, which results in higher values of power output. MFCs inoculated with sludge outperformed the rest, reaching a maximum power output of 40.38 mW·m^−2^_anode_ (1.21 mW). Results of the microbial community analysis begin to shed some light on the synergistic activities of bacterial populations, whilst metabolising a complex substrate such as urine and transferring electrons as part of anaerobic respiration.

## Figures and Tables

**Table 1:** Summary of the maximum power output by the MFC over time.

	Maximum Power Output (mW/m^2^_anode_)		
Time (Days)	Stored Urine	Sludge	Effluent
15	21.40 ± 2.5	28.95 ± 2.8	27.00 ± 3.5
30	29.64 ± 2.3	34.63 ± 1.9	33.25 ± 2.8
60	21.04 ± 3.9	31.11 ± 3.5	22.06 ± 2.1
90	21.01 ± 4.2	40.38 ± 3.7	22.61 ± 2.3
